# Association of ulcerative colitis and acute gastroenteritis with prostate specific antigen: results from National Health and Nutrition Examination Survey from (2009 to 2010) and Mendelian randomization analyses

**DOI:** 10.3389/fnut.2023.1265014

**Published:** 2023-12-04

**Authors:** Hongxiao Li, Jiefang Zheng, Weimin Dong, Yiqiao Huang, Zhengming Su, Xianhan Jiang

**Affiliations:** Key Laboratory of Biological Targeting Diagnosis, Department of Urology, Therapy and Rehabilitation of Guangdong Higher Education Institutes, The Fifth Affiliated Hospital of Guangzhou Medical University, Guangzhou, Guangdong, China

**Keywords:** ulcerative colitis, acute gastroenteritis, prostate specific antigen, NHANES, cross-sectional research

## Abstract

**Background:**

An increasing number of studies have demonstrated that gastrointestinal inflammation may increase prostate cancer risk and raise the prostate-specific antigen (PSA) level. However, the association between ulcerative colitis (UC) and acute gastroenteritis (AGE) with PSA remains unclear and complicated. Herein, we evaluated the relationship between UC and AGE with PSA concentration using the National Health and Nutrition Examination Survey (NHANES) database and Mendelian randomization (MR) analyses.

**Materials and methods:**

A total of 1,234 participants fit into the study after conducting the screening based on the NHANES survey conducted from 2009 to 2010. UC and AGE were the independent variables, and PSA was the dependent variable. Weighted multiple linear regressions were utilized to estimate the association of UC and AGE with PSA concentration. To detect the causal relationship between UC and AGE with PSA, a two-sample Mendelian randomized analysis was conducted.

**Results:**

After controlling for all covariates, PSA (log2 transform) concentrations in the UC group were increased by 0.64 (0.07, 1.21). AGE was not independently associated with PSA levels after adjusting potential confounders. In patients with coronary artery disease, AGE promotes elevated PSA (log2 transform) concentrations (*β* = 1.20, 95% CI: 0.21–2.20, *p* < 0.001). Moreover, an IVW MR analysis indicated that genetically predicted UC was associated with increased PSA, and that AGE was not associated with PSA.

**Conclusion:**

This study indicated that a positive causal association exists between UC and the PSA level. However, there is no evidence to support the relationship between AGE and the PSA level.

## 1 Introduction

Gastrointestinal inflammation is a widespread disorder that affects individuals of all ages; thus, a significantly negative clinical, economic, and humanitarian effect is exerted worldwide. Acute gastroenteritis (AGE) and ulcerative colitis (UC) are characterized by the inflammation of the digestive tract. UC, an idiopathic chronic inflammatory disorder that affects the gastrointestinal tract, entails an aberrant immune response to intestinal microflora. Worldwide, tens of millions of individuals exhibit UC, and its prevalence as well as the all-cause mortality rate are gradually increasing (1). UC is more prevalent in industrialized regions, including North America and Western Europe; this notwithstanding, Asia is exhibiting an increasing incidence. Statistics report a prevalence of 286 cases per 100,000 individuals in the United States, and that of 505 per 100,000 individuals in Norway (2). AGE is defined as sudden onset diarrhea (unrelated to chronic disease, with or without nausea, vomiting, fever, or abdominal pain) that is typically occasioned by viral or bacterial infection (3, 4). Globally, AGE led to nearly 89.5 million DALYs lost and 1.45 million deaths annually (5).

Individuals with gastrointestinal inflammation may exhibit an increased risk of developing cancer, with prostate cancer (PCa) accounting for the most commonly diagnosed cancer in males and the second leading cause of cancer-related death in men worldwide (6). The prostate-specific antigen (PSA), a serine protease produced by the prostate gland, has been widely utilized in clinical practice as a screening tool for PCa. For early-stage PCa detection, serum PSA-based screening is crucial; it can potentially increase the effectiveness of treatment and reduce mortality rates (7). Although PSA is a marker for the prostate, it is not specific to cancer. PSA levels can be elevated in non-cancerous conditions such as benign prostatic hyperplasia (BPH) and prostatitis.

Several researchers have reported an association between UC and PCa (8–12). However, observations pertaining to the association between UC and PCa risk were mixed. Relatively few studies have examined the relationship between UC and AGE with PSA. Therefore, we explored the correlation of UC and AGE with PSA using the National Health and Nutrition Examination Survey (NHANES) database. Their potentially causal relationship was subsequently analyzed using two-sample MR.

## 2 Materials and methods

### 2.1 NHANES

#### 2.1.1 Study design

NHANES is a 2-year cross-sectional survey conducted in the United States by the National Center for Health Statistics, which partly constitutes the Centers for Disease Control and Prevention, and this institution collects data representative of the health and nutrition status of non-institutional U.S. citizens. A complex multi-stage probabilistic sampling design, which combined interviews, questionnaires, diet, examination, and laboratory data, was utilized; thus, the health and nutrition status of the U.S. population was assessed. To represent a national sample, in which each individual represents approximately 50,000 US residents, the survey examines samples of approximately 5,000 individuals yearly, and it considers numerous regions. The NHANES protocols were approved by the National Center for Health Statistics Ethics Review Board, and all participants signed consent forms. More detailed information pertaining to NHANES is available at https://www.cdc.gov/nchs/nhanes/.

#### 2.1.2 Participants

This study examined the relationship between UC and AGE with PSA among participants who participated in the National Health and Nutrition Examination Survey (NHANES) between 2009 and 2010. In all the participants, we excluded the following individuals: (1) female participants (*n* = 5,312); (2) participants who underwent rectal examination in the last 7 days (*n* = 16); (3) participants who underwent prostate biopsy, prostate surgery, or cystoscopy in the last 4 weeks (*n* = 11); (4) participants diagnosed with prostate infection or inflammation (*n* = 24); (5) participants diagnosed with prostate cancer (*n* = 88); (6) participants with missing UC data (*n* = 2,669); (7) participants with missing AGE data (*n* = 283); and (8) participants with missing PSA data (*n* = 900). Because only ≥40-year-old men consented to PSA testing, this analysis was limited to this age group. At the end of the screening, 1,234 of the 10,537 participants were enrolled.

#### 2.1.3 Variables

Herein, the target independent variables were UC and AGE, which were obtained from self-reported personal interview questionnaire data collected in the NHANES. AGE was determined by the following medical question: Do you have a stomach or intestinal illness with vomiting or diarrhea that started during the last 30 days? Because AGE diagnosis is determined by medical history, it is appropriate to utilize the aforementioned question to determine AGE (13). UC was defined by the participant’s answer to the following question: Has a doctor or other health professional ever told you to have ulcerative colitis? The target dependent variable was PSA, which was obtained using the Hybritech PSA method contained in Beckman Access.

With regard to covariates, continuous variables included age, BMI, creatinine, ratio of family income to poverty, CRP, glycohemoglobin, HDL, and triglyceride, whereas classification variables included race, education level, marital status, smoking status, alcohol use, high blood pressure, coronary heart disease, diabetes, and stroke. The NHANES data set provided age, race, and other covariates acquisition process.

Table 1 depicts all the variables. After all the necessary variable datasets were identified using the CDC website, they were merged into a unified dataset, and an analysis was performed. In regard to NHANES, each participant was assigned a unique identifier, referred to as a “Sequence Number (SEQN),” which was utilized to identify each sampled individual during the data collection process.

**Table 1:** Characteristics of the participants.

PSA (ng/mL)	Low-PSA	High-PSA	*P*-value
UC (%)			0.002
Yes	0.22	2.03	
No	99.78	97.97	
AGE (%)			0.912
Yes	6.16	6.01	
No	93.84	93.99	
Age (years)	50.80 ± 7.71	54.81 ± 8.05	<0.001
Race (%)			0.829
Mexican American	8.51	6.83	
Other Hispanic	3.79	3.92	
Non-Hispanic White	73.95	75.93	
Non-Hispanic Black	9.10	9.17	
Other race	4.65	4.15	
Education (%)			0.622
Less than high school	18.10	17.50	
High school	22.94	25.85	
More than high school	58.90	56.65	
Unknown	0.06	0.00	
Marital status (%)			0.584
Married or living with partner	75.63	75.89	
Widowed, divorced, separated, or never married	24.37	23.94	
Unknown	0.00	0.17	
Ratio of family income to poverty (%)	3.32 ± 1.55	3.52 ± 1.53	0.025
BMI (kg/m^2^)	29.93 ± 6.07	28.72 ± 5.41	<0.001
Smoking status (%)			0.002
Non-smoker	3.96	3.74	
Ever-smoker	35.41	44.80	
Current smoker	52.60	42.11	
Unknown	8.03	9.35	
Alcohol use (%)			0.403
None	45.92	49.76	
Moderate	33.06	30.87	
Heavy	21.01	19.38	
Unknown	45.92	49.76	
Creatinine (μmol/L)	86.55 ± 33.06	90.01 ± 50.27	0.152
Triglyceride (mmol/L)	2.11 ± 1.76	1.93 ± 1.54	0.058
HDL (mmol/L)	1.23 ± 0.39	1.26 ± 0.41	0.164
CRP (mg/dL)	0.33 ± 0.67	0.38 ± 0.93	0.316
Glycohemoglobin (%)	5.86 ± 1.13	5.74 ± 0.81	0.029
High blood pressure (%)			0.680
Yes	36.67	35.46	
No	63.23	64.54	
Unknown	0.09	0.00	
Coronary heart disease (%)			0.253
Yes	4.59	4.96	
No	94.81	94.98	
Unknown	0.60	0.06	
Diabetes (%)			0.527
Yes	11.89	10.59	
No	85.19	85.53	
Unknown	2.93	3.87	
Stroke (%)			0.026
Yes	1.30	3.62	
No	98.67	96.28	
Unknown	0.03	0.10	

Mean + SD for continuous variables: *P*-value was calculated by weighted linear regression model. % for Categorical variables: *P*-value was calculated by weighted chi-square test.

#### 2.1.4 Statistical analysis

We utilize R^1^ and EmpowerStats^2^ for all statistical analysis. Using the NHANES analysis guidelines, weights were considered. Means ± standard were calculated for continuous variables, and percentages were calculated for categorical variables. Due to the high right-skewed PSA distribution, a log2 transformation was utilized for analysis. To estimate the association of UC and AGE with PSA concentration, we adopted weighted univariate and multiple linear regression models; thus, we constructed three statistical models, namely Model 1, Model 2, and Model 3. In Model 1, no covariates were adjusted; in Model 2, demographic data, including age, race, education, marital status, and PIR were adjusted; and in Model 3, all the covariates presented in Table 1 were adjusted. Moreover, subgroup analyses and interaction tests were performed. All tests were considered statistically significant at *p* < 0.05.

### 2.2 Mendelian randomization study

#### 2.2.1 Study design

To investigate the causal relationships between AGE, UC, and PSA, we conducted a two-sample MR study. Herein, two-sample MR represents a method for identifying the causal relationship between the exposure phenotype and the outcome; this method entails utilizing the genetic variants for the exposure as the instrument variables, which could leverage the publicly available dataset from large sample genome-wide association studies (GWAS) and compensate for the shortcomings typical of observational studies.

#### 2.2.2 Summary dataset of exposures and outcome

The UC genetic association data was obtained from a genome-wide association study (GWAS) analysis of 6,968 UC cases and 20,464 controls of European ancestry (GWAS ID: ieu-a-32) conducted by the IIBDGC consortium (14). In this dataset, 27,432 European (6,968 UC cases and 20,464 controls) were analyzed, and 12,255,197 single-nucleotide polymorphisms (SNPs) were identified. We utilized the presumed infectious origin summary data pertaining to diarrhea and gastroenteritis. The data, which was obtained from the FinnGen research project, comprised 26,288 cases and 305,879 control, and represents acute gastroenteritis. Outcome data were obtained from a publicly available GWAS dataset (GWAS ID: prot-a-1661) (15), and this dataset contained 3,301 Europeans with 10,534,735 SNPs.

#### 2.2.3 SNP selection

First, SNPs were selected at a threshold of genome-wide significance (*p* < 5 × 10–7). Second, suitable SNPs were retained based on linkage disequilibrium as measured by r2 > 0.01 in a clumping algorithm with a cut-off value. Using the preceding approach, we screened 64 SNPs associated with UC and 7 SNPs associated with AGE.

#### 2.2.4 Statistical analysis

A two-sample MR analysis between exposures and outcomes was performed using the R package “TwoSampleMR” (version 0.5.6). The inverse-variance weighted (IVW, random effects) method was utilized for primary analysis, and the MR-Egger and weighted median methods were utilized for additional analysis. When the assumption that all included SNPs can be utilized as efficient IVs is satisfied, the IVW method provides a precise estimate. MR-Egger regression can detect and adjust for pleiotropy; however, the accuracy of the produced estimate is quite low (16). The weighted median provides an accurate estimate based on the assumption that at least 50% of the IVs are valid (17). Subsequently, we utilized Cochran’s Q tests to evaluate the heterogeneity between the SNPs included in each analysis, and we utilized the MR-Egger regression to evaluate potential directional pleiotropy by testing for the intercept term. This indicates that directional pleiotropy might not exist when the intercept term approximates zero (18). To judge the stability of the MR results, the leave-one-out sensitivity test was utilized by excluding IVs one at a time (19). In addition, to assess the horizontal pleiotropy level of IVs, MR-PRESSO was utilized (20).

## 3 Results

### 3.1 NHANES

A total of 1,234 cancer-free male adults aged between 40 and 69 years were enrolled. The baseline characteristics of the participants as per the PSA median were depicted in Table 1. Compared to low-PSA participants, high-PSA participants were older, exhibited significantly higher family income to poverty ratio levels, and reported higher UC and stroke incidences. By contrast, low-PSA participants exhibited higher BMI and glycohemoglobin levels.

The results of the univariate and multivariate analyses, conducted by the weighted multivariable linear model, were depicted in Table 2. Model 1, an unadjusted model, indicated that PSA (log2 transform) concentrations in the UC group increased by 0.78 (0.19, 1.36), compared with the non-UC group. Model 2 was a minimally adjusted model. After adjusting for age, race, education level, marital status, and ratio of family income to poverty, PSA (log2 transform) concentrations in the UC group increased by 0.68 (0.10, 1.25), compared with the non-UC group. The fully adjusted model adjusts for age, race, education level, marital status, ratio of family income to poverty, BMI, smoking status, alcohol use, creatinine, CRP, glycohemoglobin, HDL, triglyceride, high blood pressure, coronary heart disease, diabetes, and stroke. Compared with the non-UC group, PSA (log2 transform) concentrations in the UC group increased by 0.64 (0.07, 1.21). The positive correlation between UC and PSA existed in all three models. Results obtained from the weighted linear regression indicated that there was no significant relationship between AGE and PSA across all these three models.

**Table 2:** The association between UC and AGE with PSA.

	Non-adjusted model β (95% CI) *P*-value	Minimally-adjusted model β (95% CI) *P*-value	Fully-adjusted model β (95% CI) *P*-value
PSA			
Non-UC	Reference	Reference	Reference
UC	0.78 (0.19, 1.36) 0.009	0.68 (0.10, 1.25) 0.021	0.64 (0.07, 1.21) 0.029
Non-AGE	Reference	Reference	Reference
AGE	−0.15 (−0.41, 0.11) 0.249	−0.15 (−0.40, 0.10) 0.254	−0.11 (−0.36, 0.14) 0.400

Model 1: No covariates were adjusted. Model 2: age, race, education level, marital status, ratio of family income to poverty were adjusted. Model 3: age, race, education level, marital status, ratio of family income to poverty, BMI, smoking status, alcohol use, creatinine, CRP, glycohemoglobin, HDL, triglyceride, high blood pressure, coronary heart disease, diabetes, stroke were adjusted.

As depicted in Table 3, we conducted an interaction and stratified analysis by age groups, race, BMI groups, high blood pressure, coronary heart disease, diabetes, and stroke; thus, we assessed the associations between UC and PSA. Subsequently, in Model 3, no statistical significance was indicated by the interaction terms that regulate the UC–PSA association. As illustrated in Table 4, we conducted interaction and stratified analysis by age groups, race, BMI groups, high blood pressure, coronary heart disease, diabetes, and stroke; thus, we assessed the AGE–PSA associations. Significant interaction was observed only for coronary heart disease (*p* < 0.001). In patients with coronary artery disease, AGE promotes elevated PSA (log2 transform) concentrations (*β* = 1.20, 95% CI: 0.21–2.20, *p* = 0.023).

**Table 3:** Subgroup analyses of the association between UC and PSA.

Parameters	*N*	*β*	95% CI	*P*-value	P for interaction
Age					0.914
<60 years	849	0.52	(−0.22, 1.26)	0.169	
≥60 years	385	0.59	(−0.35, 1.54)	0.220	
Race					0.137
Mexican American	257	−1.06	(−2.32, 0.19)	0.098	
Other Hispanic	127	1.16	(−1.78, 4.09)	0.442	
Non-Hispanic White	569	0.84	(0.08, 1.59)	0.030	
Non-Hispanic Black	237	−2.97	(−6.43, 0.49)	0.094	
BMI					0.838
<25 kg/m^2^	264	0.20	(−1.30, 1.70)	0.794	
25−30 kg/m^2^	494	0.72	(0.02, 1.42)	0.046	
≥30 kg/m^2^	476	0.41	(−0.91, 1.72)	0.546	
High blood pressure					0.777
Yes	489	0.64	(0.12, 1.41)	0.100	
No	744	0.73	(−0.13, 1.59)	0.097	
Coronary heart disease					0.546
Yes	67	1.13	(−3.99, 6.25)	0. 667	
No	1162	0.67	(0.10, 1.23)	0.022	
Diabetes					0. 862
Yes	183	0.52	(−0.84, 1.88)	0.454	
No	1013	0.57	(−0.06, 1.20)	0.074	
Stroke					0.546
Yes	41	2.79	(−1.99, 7.57)	0.273	
No	1191	0.68	(0.10, 1.25)	0.021	

Above adjusts for age, race, education level, marital status, ratio of family income to poverty, BMI, smoking status, alcohol use, creatinine, CRP, glycohemoglobin, HDL, triglyceride, high blood pressure, coronary heart disease, diabetes, stroke. In each case, the model was not adjusted for the stratification variable itself.

**Table 4:** Subgroup analyses of the association between AGE and PSA.

Parameters	*N*	*β*	95% CI	*P*-value	P for interaction
Age					0.433
<60 years	849	−0.22	(−0.51, 0.07)	0.136	
≥60 years	385	−0.02	(−0.56, 0.51)	0.928	
Race					0.952
Mexican American	257	−0. 41	(−0.94, 0.12)	0.129	
Other Hispanic	127	−0. 41	(−1.11, 0.30)	0.259	
Non-Hispanic White	569	−0.16	(−0.53, 0.21)	0.397	
Non-Hispanic Black	237	−0.21	(−0.89, 0.47)	0.548	
BMI					0.852
<25 kg/m^2^	264	−0.21	(−0.84, 0.43)	0.522	
25−30 kg/m^2^	494	−0.18	(−0.65, 0.28)	0.439	
≥30 kg/m^2^	476	−0.16	(−0.50, 0.18)	0.352	
High blood pressure					0.083
Yes	489	0.17	(−0.23, 0.57)	0.404	
No	744	−0.32	(−0.65, 0.01)	0.057	
Coronary heart disease					<0.001
Yes	67	1.20	(0.21, 2.20)	0.023	
No	1162	−0.27	(−0.53, −0.00)	0.050	
Diabetes					0.891
Yes	183	−0.12	(−0.67, 0.44)	0.682	
No	1013	−0.16	(0.45, 0.12)	0.267	
Stroke					0.064
Yes	41	0.88	(−2.15, 3.92)	0.577	
No	1191	−0.14	(−0.40, 0.11)	0.273	

Above adjusts for age, race, education level, marital status, ratio of family income to poverty, BMI, smoking status, alcohol use, creatinine, CRP, glycohemoglobin, HDL, triglyceride, high blood pressure, coronary heart disease, diabetes, stroke. In each case, the model was not adjusted for the stratification variable itself.

### 3.2 Mendelian randomization study

As depicted in Table 5, a significant causal UC–PSA association in the IVW analysis (beta = 0.048; se = 0.020; *p* = 0.014) was observed. Cochran’s Q statistic was 63.997 (*p* = 0.441), which indicates that there was no heterogeneity between IVs. MR–PRESSO testing did not reveal any outlier SNPs (*p* = 0.410). The MR-Egger intercept test exhibited no directional pleiotropy (intercept = 0.008, se = 0.009, *p* = 0.343). A leave-one-out sensitivity analysis indicated that the association between UC genetic predisposition and PSA was not considerably affected by any of the individual SNPs.

**Table 5:** Mendelian randomization analysis.

Exposures	Method	MR			Heterogeneity		Pleiotropy		
		Beta	se	*p*	*Q*	*p*	Intercept	se	*p*
UC	IVW	0.048	0.020	0.014	63.997	0.441			
	MR-Egger	0.008	0.046	0.861			0.008	0.009	0.343
	Weighted median	0.053	0.030	0.078					
AGE	IVW	0.043	0.211	0.837	6.162	0.291			
	MR-Egger	1.217	0.672	0.144			−0.074	0.041	0.143
	Weighted median	0.108	0.245	0.660					

MR analysis exhibited no correlation between AGE and PSA (beta = 0.043; se = 0.211; *p* = 0.837). Cochran’s Q statistic was 6.162 (*p* = 0.291), which indicates that there was no heterogeneity between IVs. MR–PRESSO testing did not reveal any outlier SNPs (*p* = 0.350), and the robustness of the results was confirmed by the leave-one-out sensitivity test. The MR-Egger intercept test exhibited no directional pleiotropy (intercept = −0.074, se = 0.041, *p* = 0.143). A leave-one-out sensitivity analysis indicated that the overall result may be altered when removing rs73410764.

**Figure 1:**
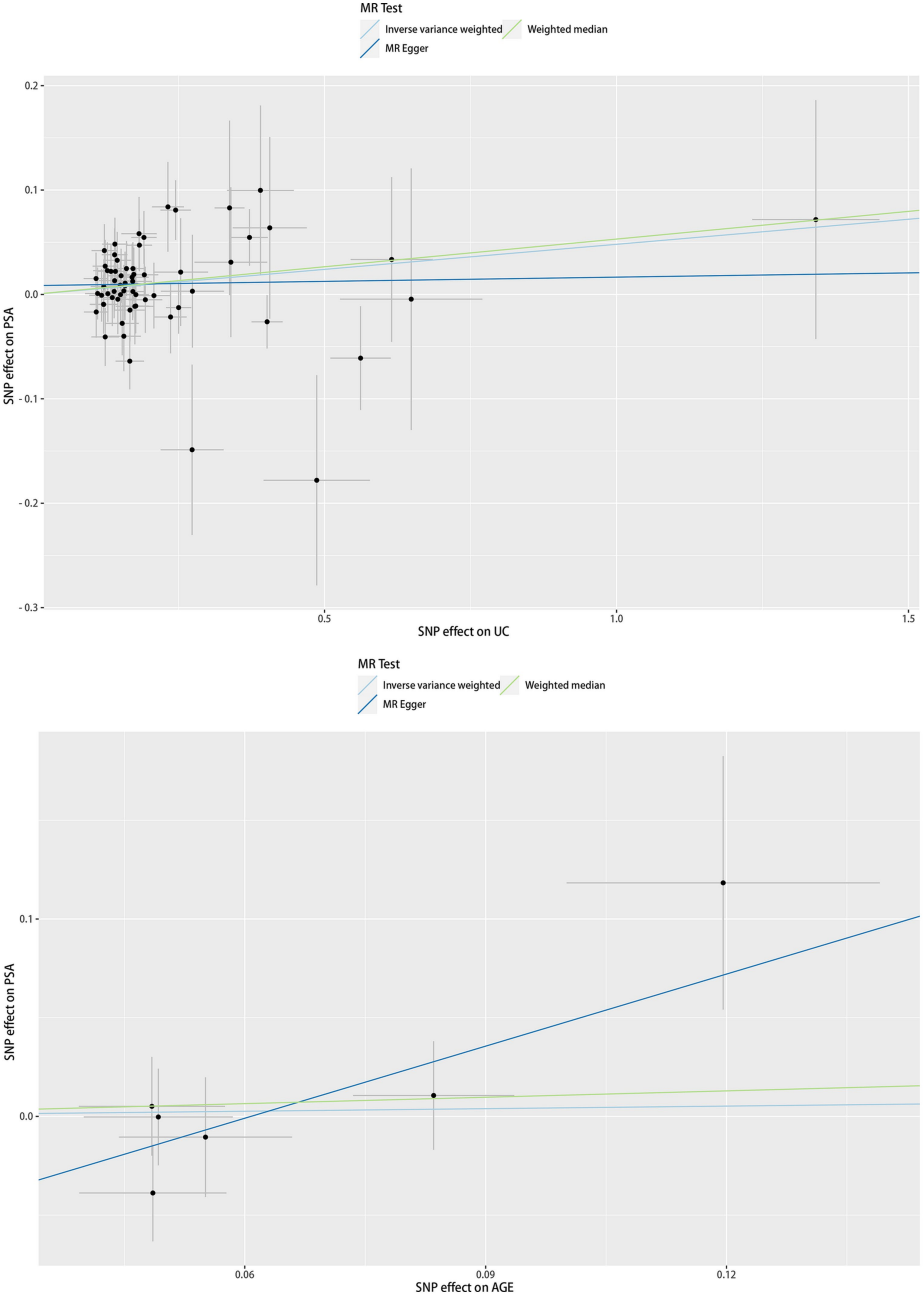
Scatter plots of causality. Along with the slope of each line corresponding to the effect of MR estimated under different models.

**Figure 2:**
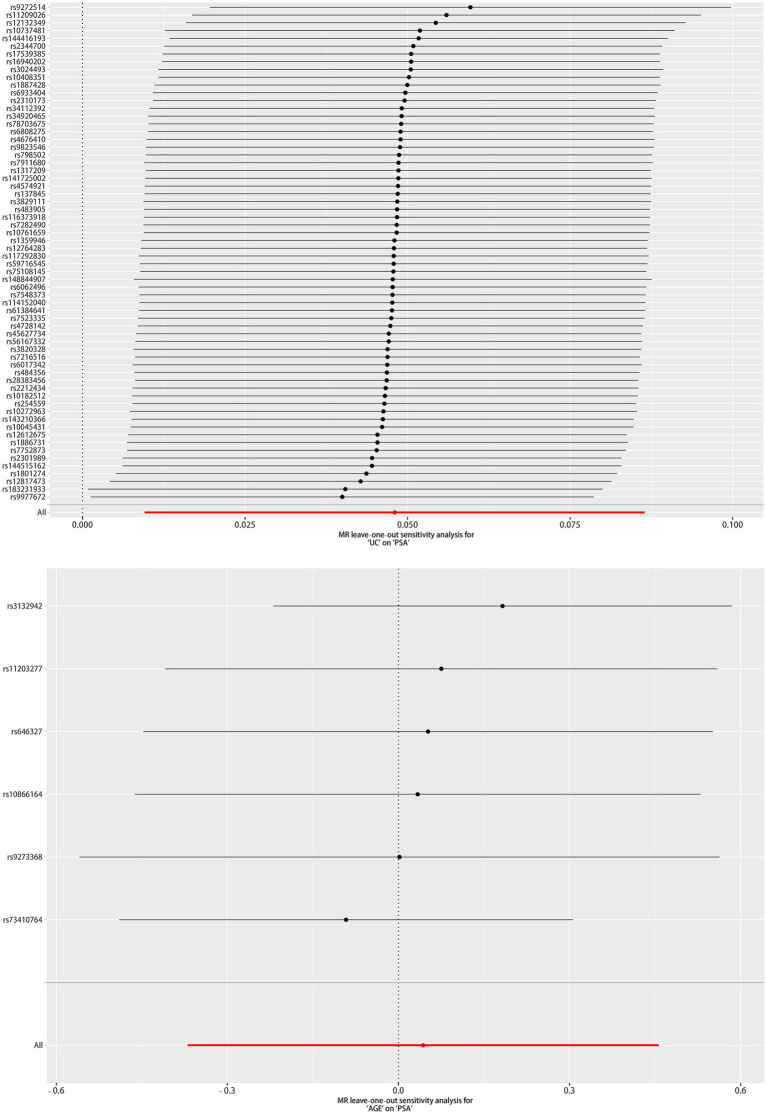
Leave one out of sensitivity tests. After removing the IVs one by one, compute the MR results for the remaining IVs.

The scatter plot of the causal relationships between UC and AGE with the risk of PSA is illustrated in Figure 1, and the sensitivity analyses details are depicted in Figure 2 and Table 5.

## 4 Discussion

The reviewed literature indicates that this pioneering study examines the relationship between UC and AGE with serum PSA concentrations based on the NHANES database and Mendelian randomization analysis. By performing an analysis of 1,234 NHANES participants, we observed that for men, UC participants exhibited higher PSA levels compared to non-UC ones. Moreover, we performed a stratified analysis, and the results have not been significantly altered; thus, the associations are robust. Furthermore, the MR analysis indicated the causal relationships between UC and PSA. Overall, no association was observed between AGE and PSA levels among men. However, using stratified analysis and interaction tests, we noted that AGE contributed to PSA elevation in the coronary heart disease population. Moreover, the MR analyses did not support causal relationships between AGE and PSA.

With respect to evaluating prostate cancer, the PSA test is currently the most widely utilized noninvasive tumor marker; however, PSA is not exclusively expressed by malignant tissues. In regard to serum, the increase in PSA levels is occasioned by the disruption of prostate gland microarchitecture, which enables PSA to cross into the extracellular space surrounding the gland. With the exception of prostate cancer, inflammation of the prostate, urinary retention, ejaculation, and ambulation influence the PSA value (21, 22).

Research into the association of UC with PCa has been limited, and, where available, such research has exhibited mixed results. Several studies have noted that UC does not promote prostate cancer (11, 23). In addition, several systematic reviews and meta-analyses have indicated that, in comparison with patients without UC, those with UC are more likely to develop cancer (24–26). Although numerous epidemiologic studies have observed relationships between UC and PCa, fewer studies have examined the UC–PSA relationships. Burns et al. documented increased PSA levels in the IBD group, and a mixed-effect regression model revealed the PSA–IBD association; however, because this study evaluated only patients presenting at an academic medical center, its external validity is limited (27).

Gut microbiota is involved in the progression of various human malignancies, including PCa. Dysbiosis of the gut microbiota crucially affects UC pathogenesis (28). Research indicates that gut dysbiosis promotes prostate cancer progression by activating the NF-κB-IL6-STAT3 axis (29). It is possible that the underlying mechanism delineating the UC–PSA association concentration is effected through the NF-κB-IL6-STAT3 pathway, and that it entails modifying the gut microbiota. There is a production dysregulation of numerous inflammatory mediators including IL-6 and IL-8 in patients with UC and in various UC experimental models (30, 31). Research has revealed that IL-6 and IL-8 correlate with increasing PSA levels (32). We propose that inflammatory mediators may partly account for the causal UC–PSA association. An existing study has indicated that chronic intestinal inflammation is associated with the increased prostatic expression of pro-inflammatory cytokines TIMP1, RANTES, and CXCL1 (33). Due to the high levels of the aforementioned factors, the activation of AKT and NF-κB pathways (34–36), which crucially affect prostate cancer development, occurs. The pro-inflammatory pathogenic microorganisms that occasion UC are introduced into the prostate through the urinary tract or circulatory system, which in turn stimulates prostate inflammation, thereby occasioning elevated PSA (37). Not only Pca and prostate inflammation but also prostate enlargement contribute to elevated PSA. The previous study that we conducted revealed that UC induced prostate enlargement (38).

The current study exhibits the following advantage. Because the study utilizes nationwide survey data, weighted data were utilized in all analyses; thus, the conclusions can be more easily generalized to the US population. In addition, a wide range of potential confounders were adequately adjusted to ensure an independent association between UC and AGE with serum PSA concentrations. In addition to the observational study based on the nationally representative NHANES, we included two-sample causal MR analyses; thus, the observations are more credible and reliable. Nevertheless, the current study exhibits some limitations, which should be considered when interpreting the results. First, due to the limited availability of UC data, the NHANES study data obtained from this study considered only the 2009–2010 period; therefore, more recent NHANES study data cannot be utilized. Second, it is probable that information bias such as information on the UC and AGE that was assessed through a questionnaire, which could affect the association, was observed.

## 5 Conclusion

By applying two different methods, the current study provided valid evidence to support the following notion: UC promotes elevated PSA levels, and AGE does not promote elevated PSA. On the other hand, in the population with coronary heart disease, UC promotes the increase of serum PSA levels.

## Data availability statement

Publicly available datasets were analyzed in this study. This data can be found at: https://www.cdc.gov/nchs/nhanes/.

## Ethics statement

The studies involving humans were approved by NCHS Research Ethics Review Board. The studies were conducted in accordance with the local legislation and institutional requirements.

## Author contributions

HL: Methodology, Writing – original draft. JZ: Writing – original draft, Methodology. WD: Supervision, Validation, Writing – review & editing. YH: Writing – review & editing. ZS: Writing – review & editing. XJ: Methodology, Writing – review & editing.

## Footnotes

^1^ http://www.R-project.org

^2^ http://www.empowerstats.com
